# A practical model for the train-set utilization: The case of Beijing-Tianjin passenger dedicated line in China

**DOI:** 10.1371/journal.pone.0175698

**Published:** 2017-05-10

**Authors:** Yu Zhou, Leishan Zhou, Yun Wang, Xiaomeng Li, Zhuo Yang

**Affiliations:** 1 MOE Key Laboratory for Urban Transportation Complex System Theory and Technology, School of Traffic and Transportation, Beijing Jiaotong University, Beijing, China; 2 Department of Civil, Environmental, and Infrastructure Engineering, Volgenau School of Engineering, George Mason University, Fairfax, Virginia, United States of America; Beihang University, CHINA

## Abstract

As a sustainable transportation mode, high-speed railway (HSR) has become an efficient way to meet the huge travel demand. However, due to the high acquisition and maintenance cost, it is impossible to build enough infrastructure and purchase enough train-sets. Great efforts are required to improve the transport capability of HSR. The utilization efficiency of train-sets (carrying tools of HSR) is one of the most important factors of the transport capacity of HSR. In order to enhance the utilization efficiency of the train-sets, this paper proposed a train-set circulation optimization model to minimize the total connection time. An innovative two-stage approach which contains segments generation and segments combination was designed to solve this model. In order to verify the feasibility of the proposed approach, an experiment was carried out in the Beijing-Tianjin passenger dedicated line, to fulfill a 174 trips train diagram. The model results showed that compared with the traditional Ant Colony Algorithm (ACA), the utilization efficiency of train-sets can be increased from 43.4% (ACA) to 46.9% (Two-Stage), and 1 train-set can be saved up to fulfill the same transportation tasks. The approach proposed in the study is faster and more stable than the traditional ones, by using which, the HSR staff can draw up the train-sets circulation plan more quickly and the utilization efficiency of the HSR system is also improved.

## Introduction

During the past few years, China has been in a period when high-speed railway (HSR) developed rapidly, with the operation mileage increasing from 404 kilometers in 2008 to over 19 thousand kilometers at the end of 2015. With the gradually formed HSR network, the mobility of society, including people and cargo, is increasingly dependent on the HSR. Even though the imbalance between transport supply and demand has been slightly mitigated by introducing HSR, great efforts are still needed to improve the transport capability of HSR, so as to satisfy the explosively increasing mobility demand. Generally, the transport capability of HSR can be improved from two aspects. Firstly, speeding up the infrastructure construction can raise the upper bound of capacity. Secondly, improving the operation and management of HSR is an effective way to make the most of the existing infrastructure. Considering the long construction period, it is impractical to increase the HSR operation mileage within a short time. Therefore, enhancing the utilization efficiency of HSR is an achievable and key solution to improve the transport capacity and mitigate the social contradiction of imbalance between transport supply and demand.

As the carrying tools of HSR, train-sets play a vital role in the process of completing society mobility task, the utilization efficiency of which is one of the most important factors influencing the transport capacity of HSR. Therefore, how to improve the train-set utilization efficiency has been a research hotspot in railway transport for several decades. The utilization efficiency of train-sets is directly dependent on the train-set utilization plan, which is made in a given train graph by a number of train-sets to complete the trip tasks. When a train-set utilization plan is drawn up, the amount of train-sets in demand as well as the working time and idle time of each train-set can be obtained. Obviously, by following a good train-set utilization plan, the proportion of train-sets’ working time in each day can be dramatically increased. Thus, a number of train-sets can be saved to serve for other train schedules, which means more trips can be added to the train graph without considering the limitation of available train-sets.

During the past few decades, extensive research has been carried out worldwide to make an attempt to draw up a high-quality train-set utilization plan, leading to the development of various models and techniques. In Europe, the train-set utilization optimization problem mainly focused on the coupling and uncoupling arrangements of train-sets to carry out the trip tasks in the train graph. Schrijver [1] firstly paid attention to the train-set scheduling problem. He proposed a model based on the minimum cost flow theory and solved it by the software CPLEX. Abbink et al. [2] explored the train marshalling problem during the peak period, and the optimization goal was set to minimize the shortage number of train seats. Alfieri et al. [3] addressed the train-set utilization problem on a single train line in a single day. An integer programming model was described to obtain the circulation of rolling stock considering the order of the train units in the compositions. Fioole et al. [4] put forward a mixed integer programming model on basis of previous research achievements, and the improved branch and bound algorithm was applied to obtain the optimization solution. Barnhart et al. [5], Peeters and Kroon [6], and Holmberg & Yuan [7] used the Dantzig-Wolfe decomposition approach [8] to solve the train-set circulation problem, instead of the classical flow decomposition method used in other network flow problems. Maróti and Kroon [9] proposed an integer programming model with the consideration of maintenance constraint. Moreover, he designed a heuristic algorithm to solve the model and obtained a feasible solution. However, the operational mode of train-sets in China is quite different from that in European countries. In China, the train-sets are used as a whole, so there is no need to consider the problem of coupling and uncoupling. Zhao at el. [10] discussed the different utilization modes of train-sets comprehensively and they found that the mode of unrestrained section can improve the utilization efficiency. Nie et al. [11] proposed an integer programming model considering the deadhead trains condition, and applied the Hungary algorithm to solve the problem. Wang et al. [12] set up a train-set utilization model to complete the trip tasks in a cyclic train timetable, and a column generation algorithm was used to obtain the optimal solution. Then Wang et al. [13] improved this model, and designed a method of branch and price, and the findings have inspired latter research. Zhao [14] believed that the train-set utilization problem can be regarded as a typical NP hard problem, and it cannot be solved directly by ready-made software. Characterized by easy-to-understand and fast, a large number of heuristic algorithms have been applied to solve the train-set utilization problem. Yang et al. [15] built a train-set connection network model aiming to maximize the train-set utilization efficiency, and designed a genetic algorithm to solve the model. Chen et al. [16] proposed a multi-objective optimization model to maximize the equilibrium degree of train-set utilization. Considering the number of train-sets in use, a simulated annealing algorithm was designed to solve the model. Hong et al. [17] put forward an integer programming model considering the maintenance and train graph constraints, and a heuristic approach was applied to solve this problem.

Recent research on the train-set utilization problem mainly focuses on developing new methodologies and introducing rescheduling procedures. The train-set utilization problem has been proved to be a TSP (Traveling Salesman Problem) with some special constraints. Based on TSP, Zhang [18] built a train-set utilization model in a railway network by taking both the running time constraint and running distances constraint into consideration, and an ant colony algorithm was used to solve the model; Zhou [19] proposed an integer programming model to obtain the train-set utilization plan, considering the constraint of Level 1 maintenance standard, and an improved ant colony algorithm was designed to solve the model. Thorlacius [20] proposed an integrated rolling stock planning model that took into account all practical requirements simultaneously for rolling stock planning at DSB S-tog. By using a hill climbing heuristic algorithms to solve this model, the efficiency of new rolling stock plan was increased by 2%. Jørgen [21] designed two approaches, one of which is Mixed Integer Linear Program (MILP) solved by CPLEX and the other is column and row generation approach, and both approaches are sufficiently fast in real-time setting. Lai [22] put forward a hybrid heuristic process to solve this problem for Taiwan Railway Administration (TRA). The efficiency of rolling stock use was increased by about 5% and the solution time was reduced significantly from 3h to 11.2s. Tatsushi [23] proposed a column generation and Lagrangian relaxation heuristics, and good lower and upper bounds for 300 trips were found within reasonable computing time. Due to disturbances or disruptions from the real world, the train-set circulation plan need to be rescheduled in order to maintain a feasible rolling stock circulation, and generally, this procedure should be carried out in a short time. Some researchers studied the train-set utilization problem in the rescheduling procedures. Cadorso [24] proposed a new approach to obtain better and more robust circulations of the rolling stock train units, solving the rolling stock assignment while accounting for the train routing problem. By designing a heuristic based on the benders decomposition, more robust and efficient solutions were obtained. Wagenaar [25] proposed three different models for rescheduling, namely the Extra Unit Type model, the Shadow-Account model and the Job-Composition model. All models were tested on instances of Netherlands Railways. Results showed that the models were able to take maintenance appointments into account efficiently. Cacchiani [26] presented an overview of recovery models and algorithms for real-time railway disturbance and disruption management, and the application of these models and algorithms in real-life railway systems would be instrumental in improving the quality of provided railway services, leading to an increased utilization of the involved railway systems. Table 1 provides a systematic comparison of key model components and solution methods in the existing research.

**Table 1:** Comparison of key model components and solution methods in the existing train-set utilization research.

Modeling scenarios	Modeling features	Objective Function	Solution Method	Publication
MT, RD, NM, SB	IP	Minimize train-set costs	Solve by CPLEX	Scrijver [1]
ST, UUR, NM, SB	IP, AP	Minimize connection time	Hungarian Algorithm	Zhao et al. [14]
MT, NM	IP	Minimize absence of passenger	Solve by CPLEX	Abbink et al. [2]
ST, M, UFR	IP, MFP, TM	Minimize transition costs	Solve by CPLEX	Marὀti and Kroon [27]
RD, NM, UFR	IP, MFP	Minimize train-sets and running distance	Preprocess and solve by CPLEX	Alfieri et al. [3]
MT, RD, NM	MIP	Minimize absence of passenger and shunting	Improved Branch and Bound, CPLEX	Fioole et al. [4]
RD, NM, UFR	IP	Minimize absence of passenger and shunting	D-W Decomposition Algorithm	Peeters and Kroon. [6]
RD, NM	IP	Minimize absence of passenger and shunting	Heuristic Algorithm and CPLEX	Marὀti [28]
MT, M, UUR	NM, TSP	Minimize connection time	Computer Simulation	Geng et al. [29]
ST, M	IP	Minimize train-sets	Crossover heuristic Algorithm	Hong et al. [17]
M, UUR, MB	MOIP, TSP	Minimize train-sets and maintenance	Hierarchical Heuristic Algorithm	Miao et al [30]
ST, M, UUR	IP, TSP	Minimize connecting time	Improved Ant Colony Algorithm	Zhou et al. [19]
ST, M, UUR, MB	IP, TSP,	Minimize connecting time	Model Convert, Two Stage Method	Present Study

**Modeling scenarios:** ST-Single Train-Set Type; MT-Multiple Train-set Type; SB-Sing Base; MB-Multiple Base; RD-Reconnection and Decomposition; NM-No Maintenance; M-Maintenance; UUR-Using in Uncertain Region; UFR-Using in Fixed Region

**Modeling characteristics:** IP-Integer Programming; AP-Assignment Problem; SM-Simulation Model; MFP- Multi-Commodity Flow Problem; MIP- Mixed Integer Programming; TM-Transition Model; NM-Network Mode; TSP-Traveling Salesman Problem; MOIP-Multi-Objective Integer Programming

Although there is a comprehensive body of literature on the train-set utilization problem, no results of previous research can be directly used into the practical production. There are two major reasons. First, limited studies take the maintenance constraints into consideration, which is actually a key factor influencing the compilation of the train-set utilization plan. Second, even if the maintenance is considered, the solutions of models are obtained by applying heuristic algorithms, which is unstable and inaccurate. Therefore, to realize the objective that the train-set utilization plan can be generated by computers automatically and used in practice, an integer programming model considering the maintenance constraints is built to maximize the train-set utilization efficiency. Specially, a two-stage method is proposed in the model solving process in this study to obtain a unique solution. With this method, the train-set utilization plan can be obtained by a computer-generated system.

The remainder of this paper is organized as follows. Section 2 describes the basic situation of the train-set utilization problem. In Section 3, the optimization model of the train-set utilization plan is developed. Section 4 introduces the solution method of the model. In Section 5, a case study based on the Beijing-Tianjin HSR line is performed to illustrate the model application. Finally, Section 6 provides the conclusions marks.

## Problem description

The train-set utilization plan is drawn up to identify the work arrangements of train-sets according to the given train graph as well as the rules and regulations of maintenance. For train-sets, except for some necessary operations in the railway stations, the main work includes two parts: one is to complete all the trip tasks in the given train graph; and the other is to carry out maintenances. Therefore, the train-set utilization plan should figure out the following aspects: the number of needed train-sets; the specific train-set undertaking its corresponding trip; the sequence of the trips undertaken by the same train-set; and the time and location each train-set is maintained. Different train-set utilization plans can be made to complete the trip tasks in a given train graph. Therefore, in this study, the optimization problem of the train-set utilization plan is to complete all the trip tasks in a given train graph by utilizing the least train-sets on the premise of satisfying the requirements of the maintenance.

**Fig 1:**
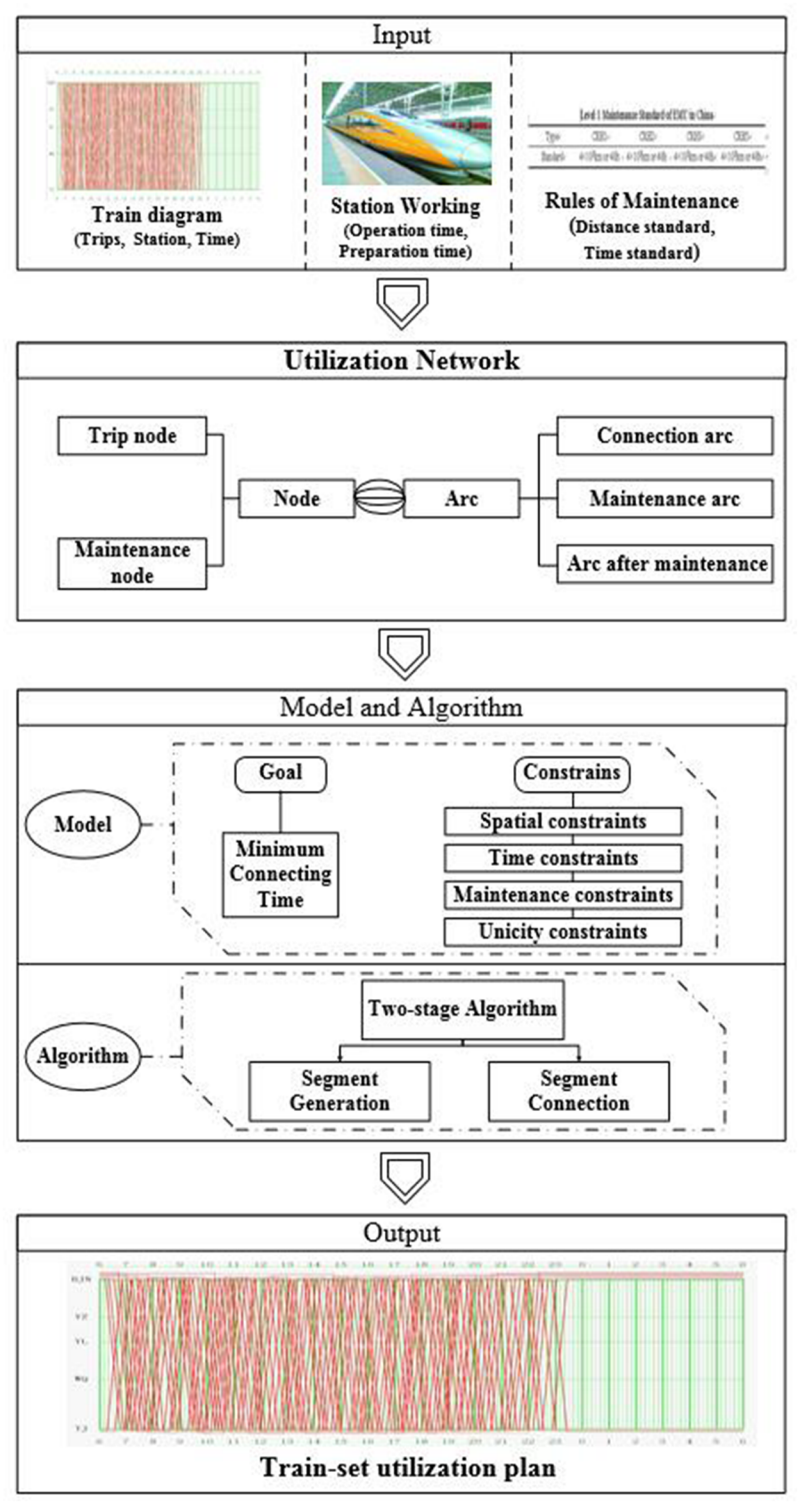
The framework of the train-set utilization optimization problem.

Fig 1 illustrates the framework of the train-set utilization optimization problem. Firstly, some basic information should be prepared as the input parameters, including: the train graph, the rules and regulations of maintenance and station working. Secondly, in order to model the train-set utilization plan, a train-set utilization network is represented by a directed graph. Thirdly, an integer programming model is established to minimize the total connecting time, considering the spatial constraints, the time constraints, the maintenance constraints, and the unicity constrains. Finally, a two-stage approach is designed to solve the problem, and an optimal train-set utilization plan is obtained as the output.

**Fig 2:**
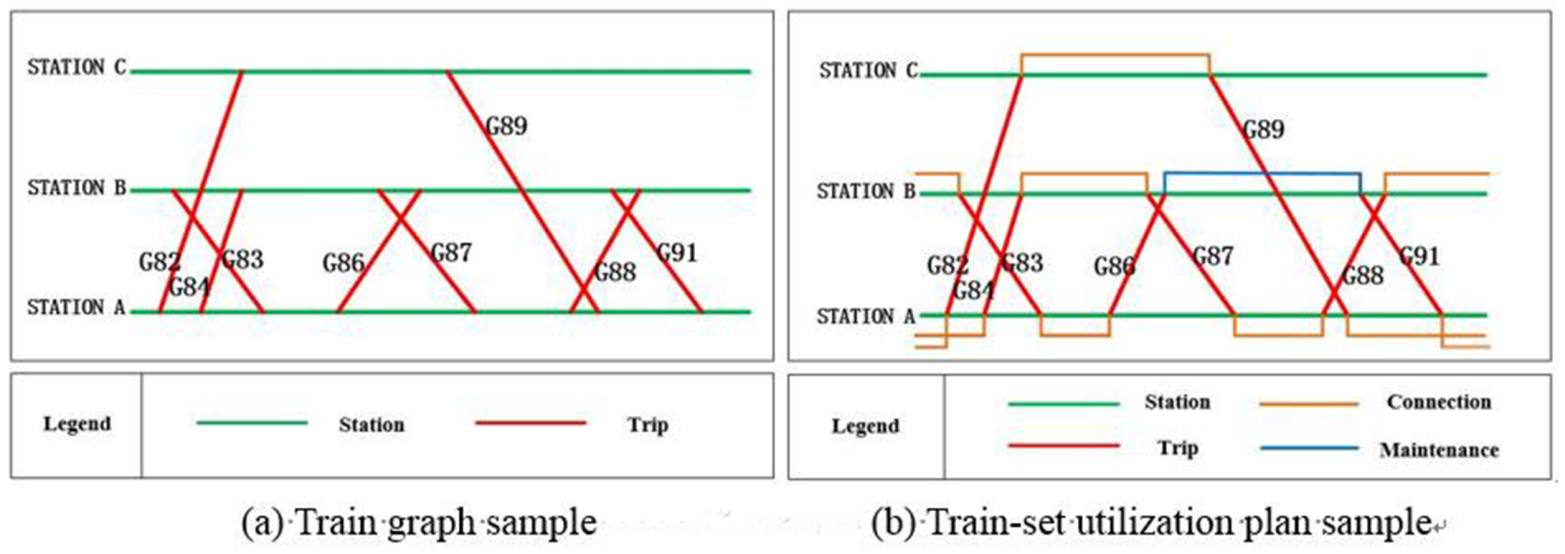
The relationship between the train graph and the train-set utilization plan.

Fig 2 illustrates the relationship between the train graph and the train-set utilization plan. Fig 2(a) is a simple sample of a given train graph, including three stations and eight trips. After adding the train-sets’ connection relationships and maintenance information, Fig 2(b) represents the train-set utilization plan. In this sample, to complete the eight transportation tasks, it needs at least three train-sets. Particularly, Trip G82 and G89 are undertaken by one train-set; Trip G84, G87, and G88 are undertaken by the second train-set; and Trip G83, G86, and G91 are undertaken by the last train-set. The train-set undertaking Trip G83 should be maintained at the inspection and repair depot near Station B after completing the task of Trip G86.

## Train-set utilization plan modeling

### Train-set utilization network representation

**Fig 3:**
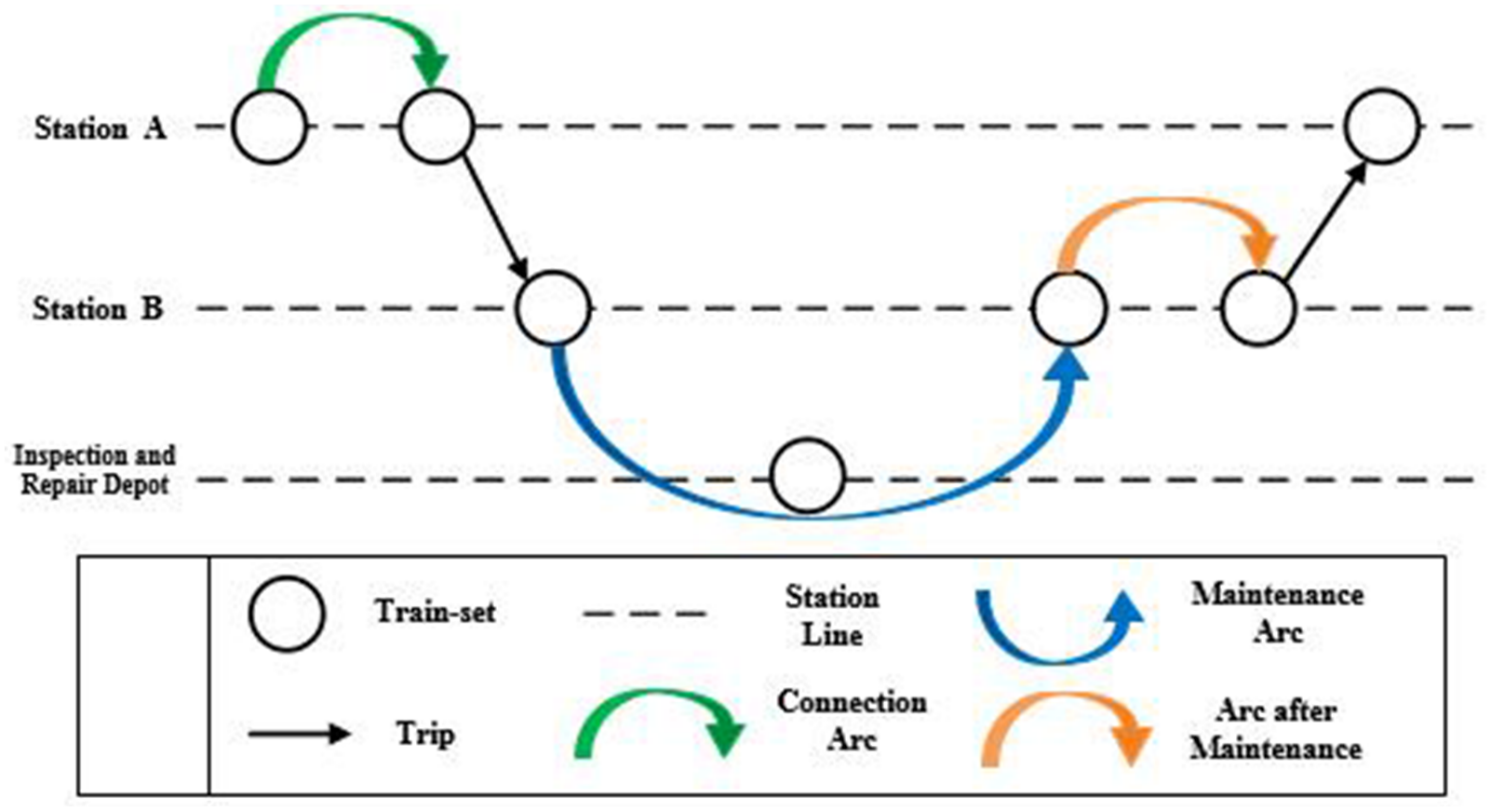
Three kinds of arcs in the train-set utilization network.

In order to model the train-set utilization plan, a train-set utilization network is represented by a directed graph *G*(*V*, *E*). The node set *V* is a union of subsets *V*_*T*_ and *V*_*M*_, which represent trips and maintenances, respectively. Each node *i* ∈ *V*_*T*_ is defined as a tuple $\left(n_{i},s_{i}^{d},s_{i}^{a},t_{i}^{d},t_{i}^{a},d_{i},t_{i}\right)$, where *n*_*i*_ indicates the train number, $s_{i}^{d}$ indicates the departure station, $s_{i}^{a}$ indicates the arrival station, $t_{i}^{d}$ indicates the departure time, $t_{i}^{a}$ indicates the arrival time, *d*_*i*_ indicates the running distance, and *t*_*i*_ indicates the running time. Each node *k* ∈ *V*_*M*_ is defined as a tuple $\left(\varphi_{k},\tau_{k}^{a}\right)$, where *φ*_*k*_ indicates the inspection and repair depot, $\tau_{k}^{a}$ indicates the time train-set departing from the inspection and repair depot to arrival station after maintenance. As shown in Fig 3, there are three kinds of arcs in the train-set utilization network, including connection arcs (i.e., trip→trip), maintenance arcs (i.e., trip→maintenance) and arcs after maintenance (i.e., maintenance→trip). Therefore, the arc set *E* consists of three disjoint sets *E*_*C*_, *E*_*Q*_ and *E*_*H*_, where *E*_*C*_ represents directional arcs between two trip nodes, i.e., connection arcs, *E*_*Q*_ represents directional arcs from trip node to maintenance node, i.e., maintenance arcs and *E*_*H*_ represents directional arcs from maintenance node to trip node, i.e., arcs after maintenance.

**Fig 4:**
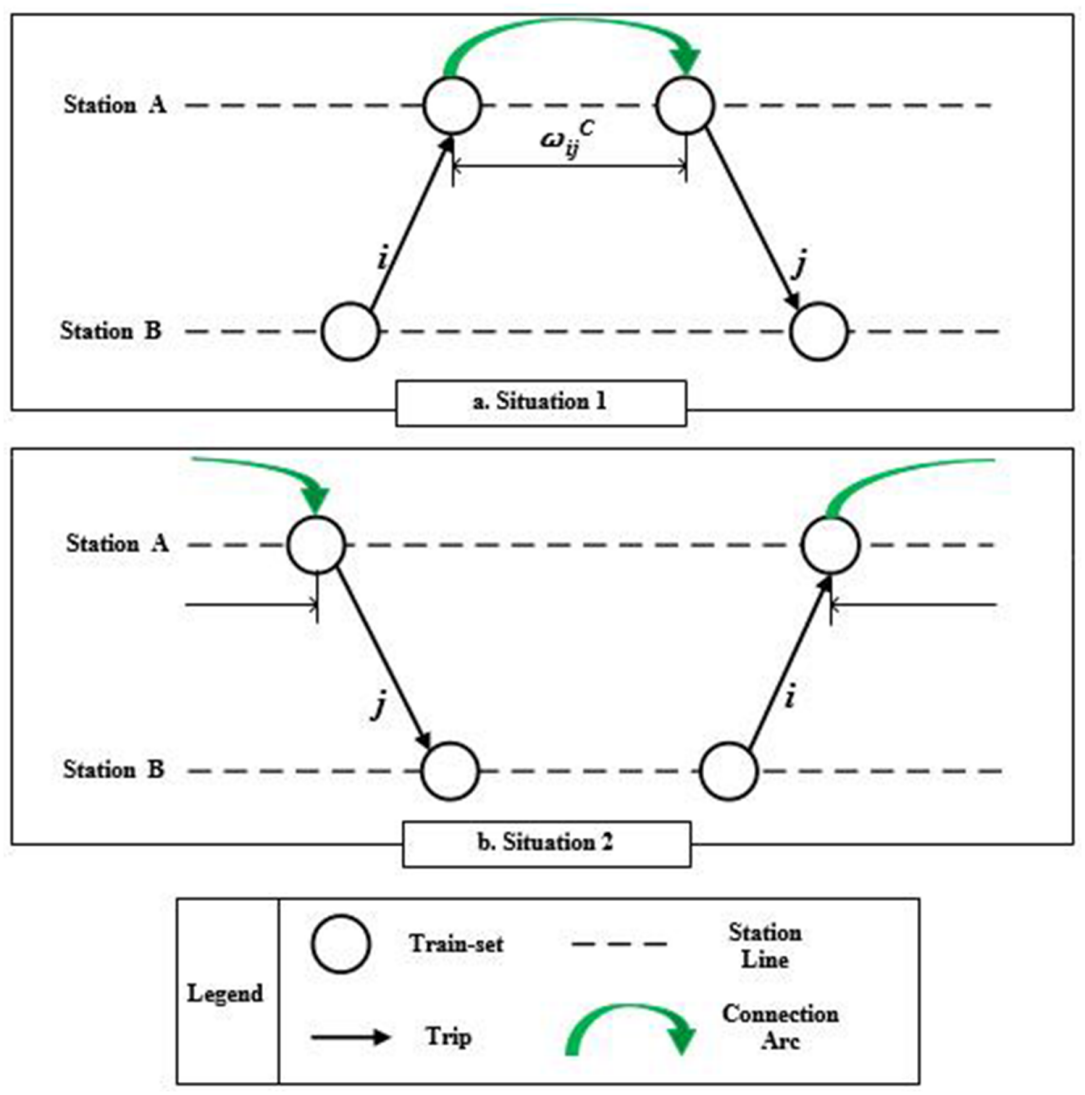
The weight of connection arcs.

**The weight of connection arcs** $\omega_{ij}^{C}$ means the connection time between Trip *i* and Trip *j*. Generally, when the arrival station of Trip *i* is different from the departure station of Trip *j*, the connection time should be defined as infinite. It means that that the two trips cannot be connected due to the huge waste of train-set empty running. As shown in Fig 4, when two trips can be connected, the values of $\omega_{ij}^{C}$ is determined by the sequential order of the two trips’ departure time and arrival time. If the departure time of Trip *j* is after the arrival time of Trip *i*, the $\omega_{ij}^{C}$ is equal to $t_{j}^{d}-t_{i}^{a}$, otherwise, the $\omega_{ij}^{C}$ is equal to $1440+t_{j}^{d}-t_{i}^{a}$, i.e., the train-set will undertake Trip *j* the next day. Based on above analyses, the values of $\omega_{ij}^{C}$ can be calculated as Eq (1).

$$\omega_{ij}^{C}=\{\begin{array}{ll} t_{j}^{d}-t_{i}^{a} & t_{j}^{d}-t_{i}^{a}\geq 0,s_{j}^{d}=s_{i}^{a}\\ 1440+t_{j}^{d}-t_{i}^{a} & t_{j}^{d}-t_{i}^{a}\leq 0,s_{j}^{d}=s_{i}^{a}\\ +\infty & s_{j}^{d}\neq s_{i}^{a} \end{array}$$ (1)

**Fig 5:**
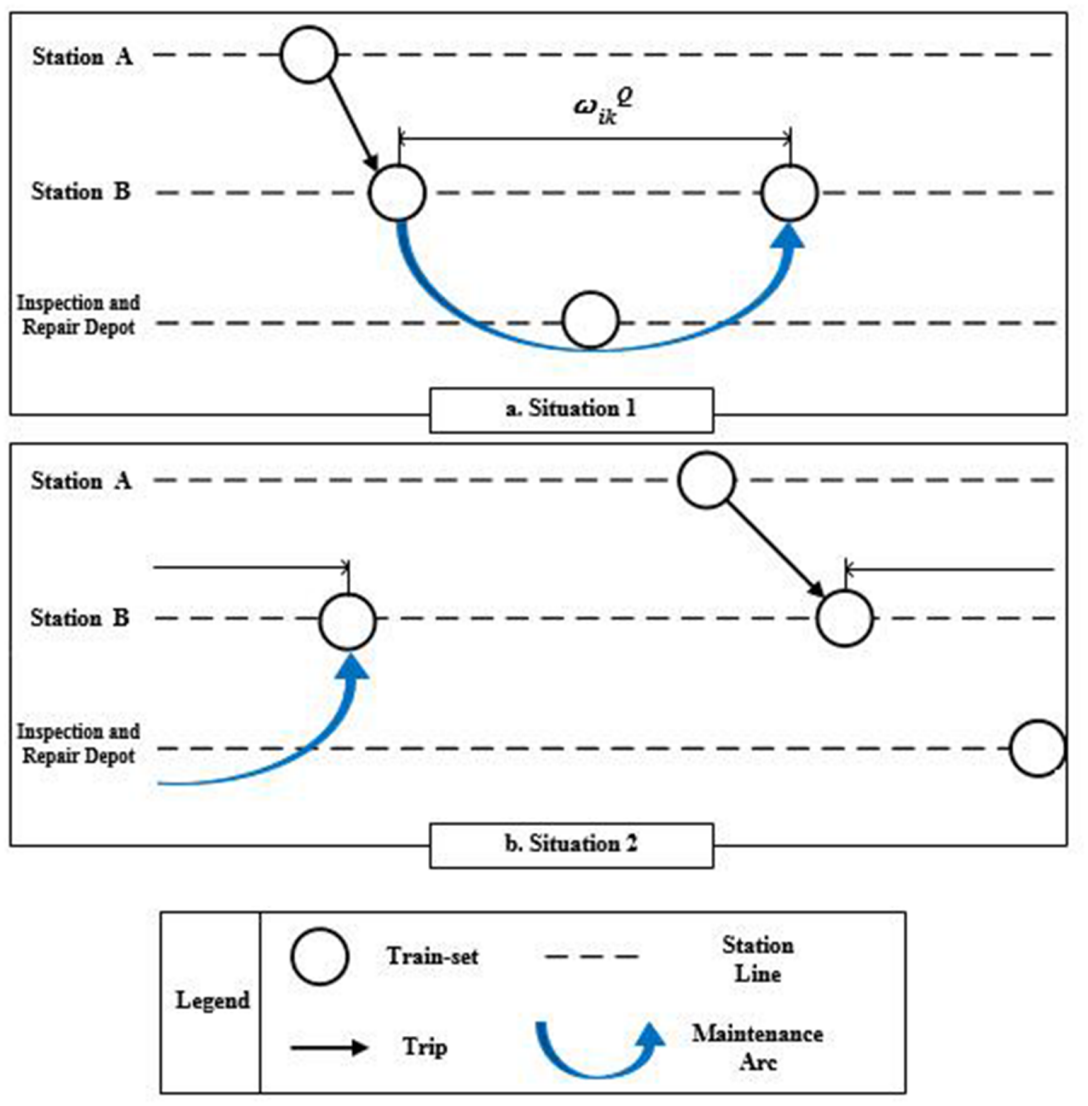
The weight of maintenance arcs.

**The weight of maintenance arcs** $\omega_{ik}^{Q}$ means the duration from the moment train-set arriving at its arrival station, $t_{i}^{a}$, to the moment train-set returning back to its arrival station after maintenance, $\tau_{k}^{a}$. As shown in Fig 5, when $\tau_{k}^{a}-t_{i}^{a}\geq 0$, the value of $\omega_{ik}^{Q}$ is equal to $\tau_{k}^{a}-t_{i}^{a}$. When $\tau_{k}^{a}-t_{i}^{a}\leq 0$, it means that the train-set can only undertake Trip *j* the next day, and the value of $\omega_{ik}^{Q}$ is equal to $1440+\tau_{k}^{a}-t_{i}^{a}$. Therefore, the values of $\omega_{ik}^{Q}$ can be calculated as Eq (2).

$$\omega_{ik}^{Q}=\{\begin{array}{ll} \tau_{k}^{a}-t_{i}^{a} & \tau_{k}^{a}-t_{i}^{a}\geq 0\\ 1440+\tau_{k}^{a}-t_{i}^{a} & \tau_{k}^{a}-t_{i}^{a}\leq 0 \end{array}$$ (2)

**Fig 6:**
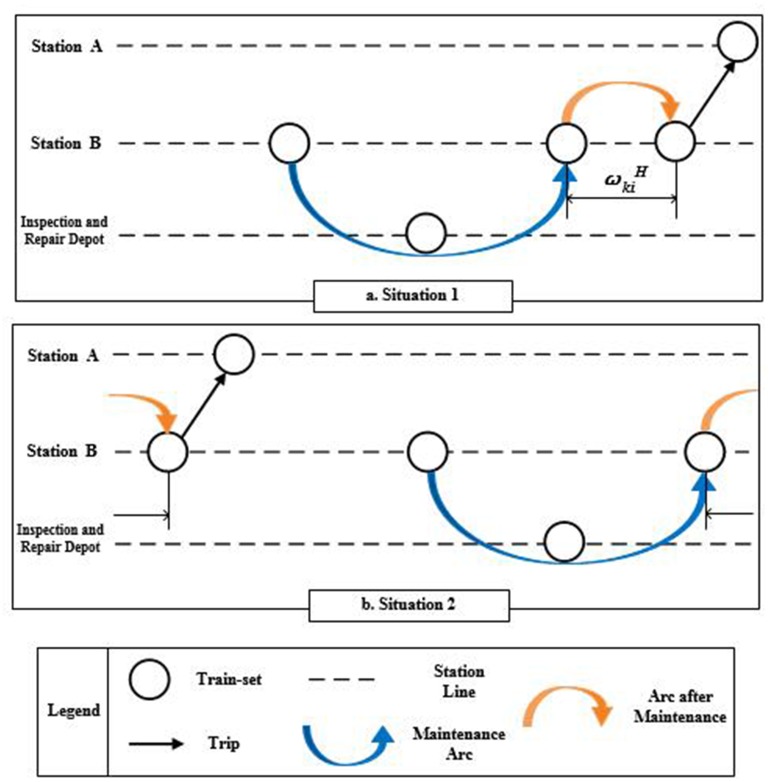
The weight of arcs after maintenance.

**The weight of arcs after maintenance** $\omega_{ki}^{H}$ means the duration from the moment train-set returning back to its arrival station after maintenance, $\tau_{k}^{a}$, to the moment train-set departing from the station, $t_{i}^{d}$. As shown in Fig 6, when $t_{i}^{d}-\tau_{k}^{a}\geq 0$, the value of $\omega_{ki}^{H}$ is equal to $t_{i}^{d}-\tau_{k}^{a}$. When $t_{i}^{d}-\tau_{k}^{a}\leq 0$, it means that the train-set can only undertake Trip *i* the next day, and the value of $\omega_{ki}^{H}$ is equal to $1440+t_{i}^{d}-\tau_{k}^{a}$. Therefore, the values of $\omega_{ki}^{H}$ can be calculated as Eq (3).

$$\omega_{ki}^{H}=\{\begin{array}{ll} t_{i}^{d}-\tau_{k}^{a} & t_{i}^{d}-\tau_{k}^{a}\geq 0\\ 1440+t_{i}^{d}-\tau_{k}^{a} & t_{i}^{d}-\tau_{k}^{a}\leq 0 \end{array}$$ (3)

### Analyses of constraints

To undertake a trip task, the train-set should satisfy four specific requirements, including the spatial constraint, the time constraint, the maintenance constraint and the unicity constraint. The detail analyses are as follows:

#### 1. Spatial constraints

As mentioned above, an empty running train-set needs to be dispatched to connect two trips. When the departure station of the latter trip is different from the arrival station of its former trip, it will cause huge waste. Therefore, as shown in Eq (4), when a train-set undertake two adjacent trips, the departure station of the latter trip, $s_{j}^{d}$, is restricted to be the same with the arrival station of the former trip, $s_{i}^{a}$, where *i* ≤ *j*.

$$s_{i}^{a}=s_{j}^{d}\;i\leq j$$ (4)

#### 2. The time constraints

**Fig 7:**
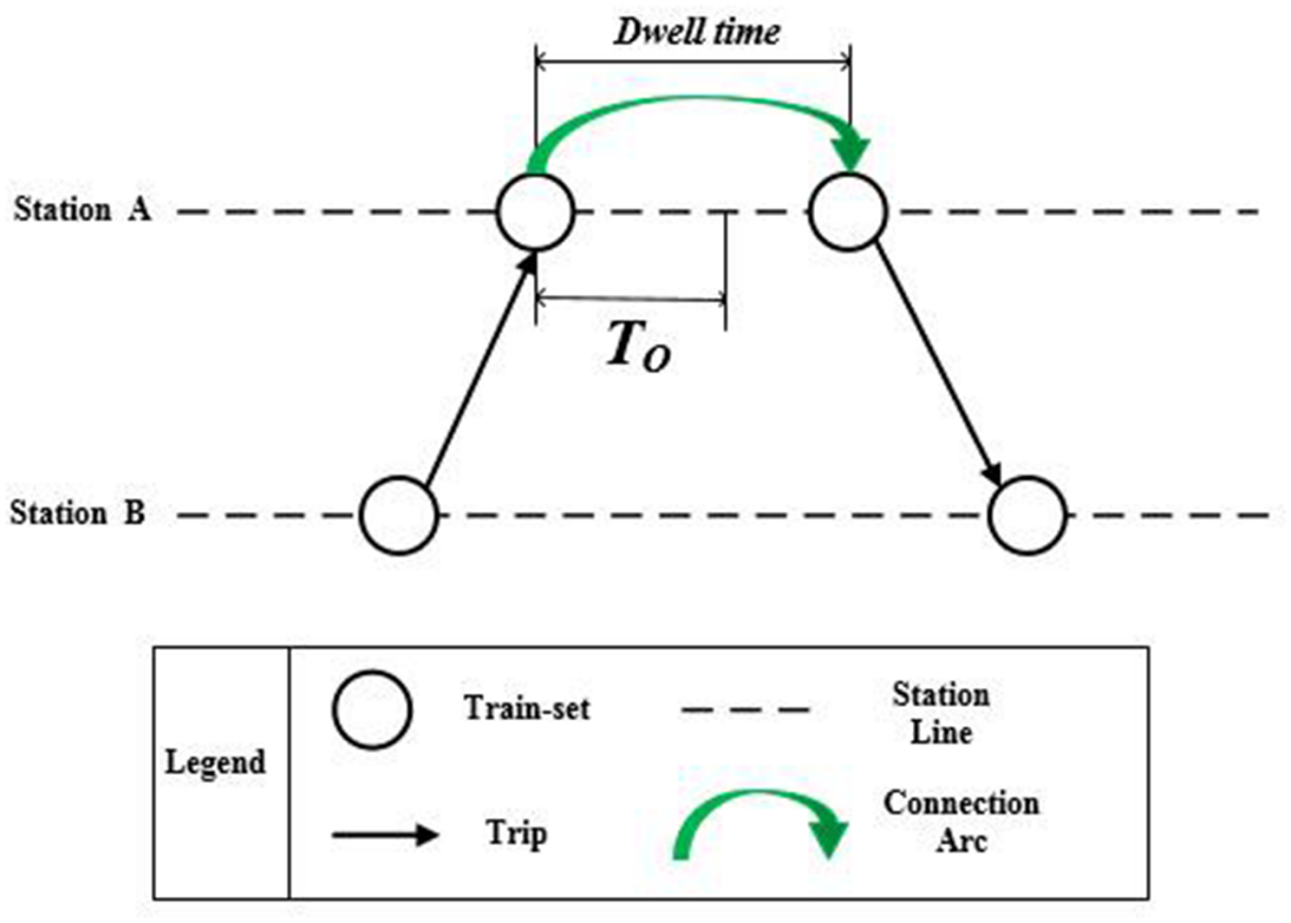
The relationship between the operation time and the weight of connection arcs.

The time constraints means that the duration of connection arcs, maintenance arcs and arcs after maintenance should respectively satisfy the requirement of the minimal necessary working procedure duration between two tasks, as shown in Fig 7.

When a train-set arrives at the station after completing the trip task, some preparation work need to be carried out before departing for the next trip, such as cleaning, pollution discharge, etc. The duration needed for such preparation work is defined as operation time, *T*_*o*_. Therefore, to ensure that two trips can be undertaken by one train-set, the connection time $\omega_{ij}^{C}$ of the two trips should be longer than the operation time *T*_*o*_, as shown in Eq (5).

$$\omega_{ij}^{C}\geq T_{o}$$ (5)

**Fig 8:**
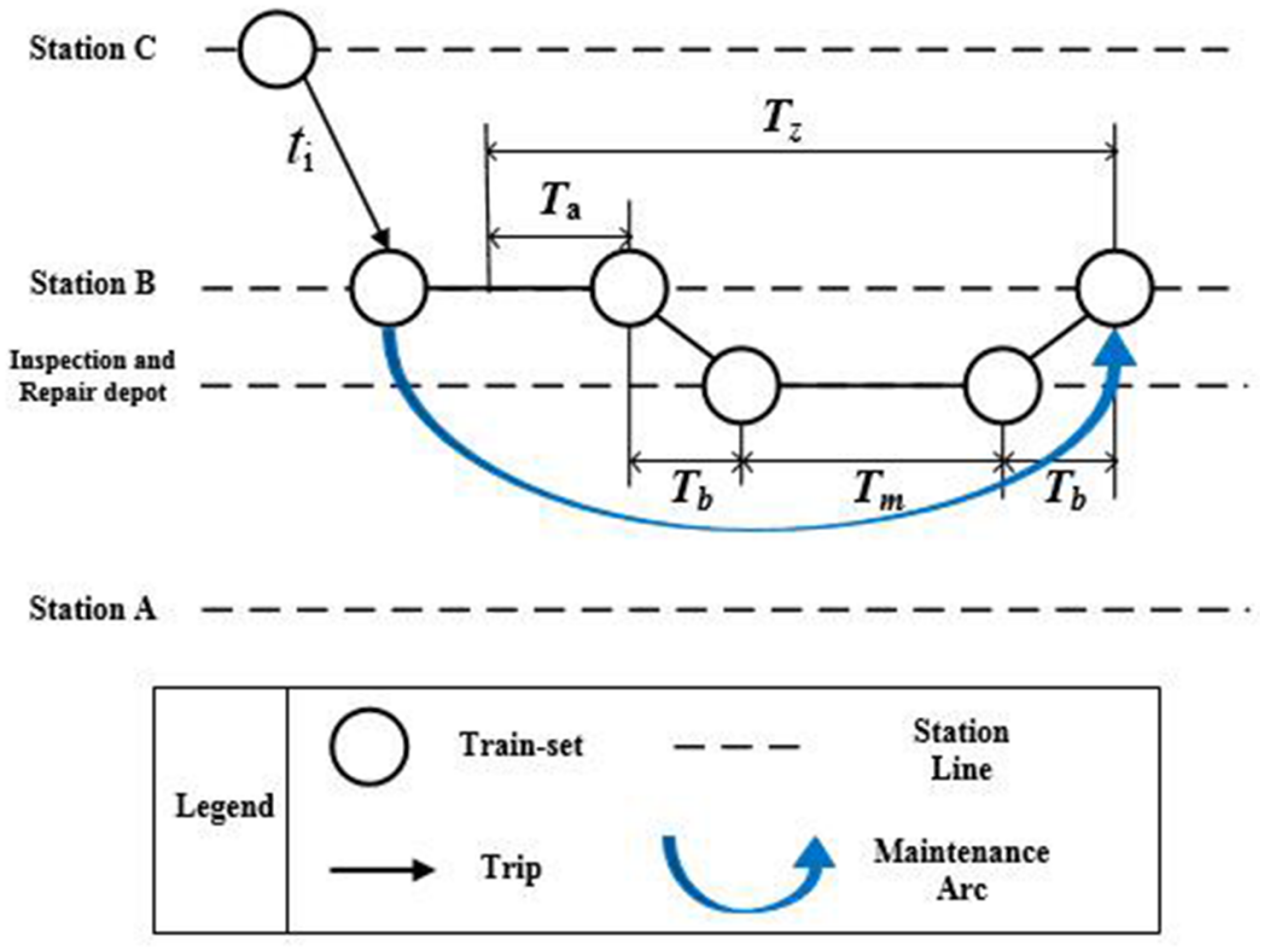
The relationship between the process duration and the weight of maintenance arcs.

When a train-set completes a trip task and reaches to the maintenance standard, it should be maintained in the inspection and repair depot. As shown in Fig 8, four essential procedures will be followed by the train-set to complete the maintenance task. Table 2 lists the detailed information about the four procedures along with their duration definitions.

**Table 2:** Four essential procedures.

Procedures		Duration	Definition
1	Some basic inspection will be carried out after the train-set arriving at the station.	Inspection time	*T*_*a*_
2	The train-set will departure from the arriving station to the inspection and repair depot.	Running time	*T*_*b*_
3	The maintenance will be carried out in the inspection and repair depot	Maintenance time	*T*_*m*_
4	The train-set will return back from the inspection and repair depot to the station.	Running time	*T*_*b*_
Total		Duration of the process	*T*_*z*_ = *T*_*a*_ + 2*T*_*b*_ + *T*_*m*_

To ensure a trip node can be connected to a maintenance node, there should be enough time for the train-set to go through these procedures. Therefore, Eq (6) restrains that the weight of maintenance arc $\omega_{ik}^{Q}$ should be larger than the duration of the process *T*_*z*_.

$$\omega_{ik}^{Q}\geq T_{z}$$ (6)

**Fig 9:**
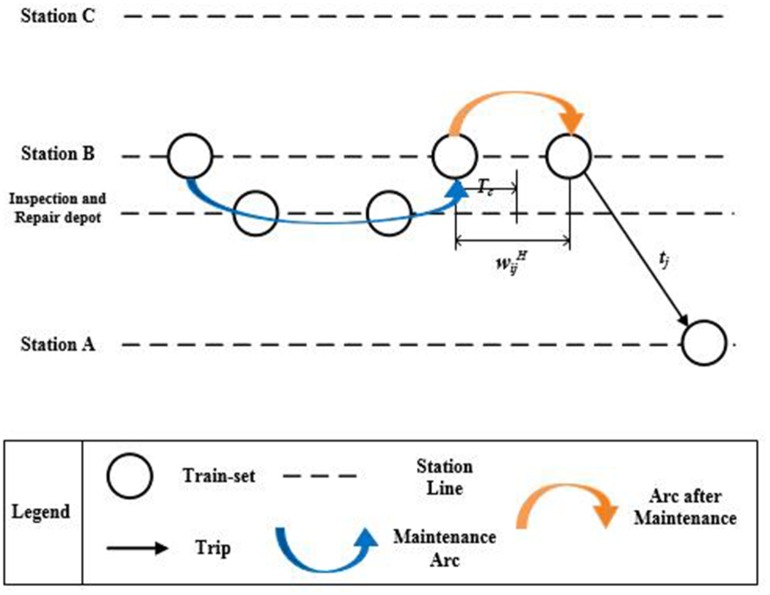
The relationship between the duration of preparation work and the weight of arcs maintenance.

Once completing the maintenance task, train-sets can be utilized to undertake the trip tasks. However, as mentioned above, a series of preparation work should be carried out before departing to ensure the train-set working normally, as shown in Fig 9. Therefore, in order to connect a maintenance node with a trip node, Eq (7) restrains that the weight value of arc after maintenance $\omega_{ki}^{H}$ should be larger than the duration of preparation work $\omega_{ki}^{H}$.

$$\omega_{ki}^{H}\geq T_{c}$$ (7)

#### 3. The maintenance constraints

For safe operation concern, the train-sets must be strictly complied with the maintenance rules and standards. In China, the train-sets’ maintenance standards are divided into five levels according to the accumulated operation time and mileages. In this study, it is assumed that the train-set utilization plan is drawn up by treating one day as a cycle. Generally, the operation time standard of Standard Level 2 to Standard Level 5 is more than a month. Therefore, only the Standard Level 1 needs to be considered in the study. The Standard Level 1 requires that when the accumulated operation time reaches up to 48 hours or the accumulated operation mileages reaches up to 4,000 km, the train-set must be maintained. Additionally, in practical applications, a 10% fluctuation compared with standard is allowable (e.g., Train-sets should be maintained when the accumulated operation mileages are within a range from 3600 km to 4400 km).

When undertaking several trips, the accumulated operation time and mileages of the train-set are $\sum_{i=1}^{n}T_{i}$ and $\sum_{i=1}^{n}d_{i}$ respectively, where *n* is the number of undertaking trips. If Δ*S*_*m*_ and Δ*S*_*t*_ represent the acceptable fluctuation ranges of operation time and mileages respectively, the accumulated operation time and mileages of each train-set should be within the range of [*S*_*t*_ − Δ*S*_*t*_, *S*_*t*_ + Δ*S*_*t*_] and [*S*_*m*_ − Δ*S*_*m*_, *S*_*m*_ + Δ*S*_*m*_], respectively.

$$S_{t}-\Delta S_{t}\leq \sum_{i=1}^{n}T_{i}\leq S_{t}+\Delta S_{t}$$ (8)

$$S_{m}-\Delta S_{m}\leq \sum_{i=1}^{n}d_{i}\leq S_{m}+\Delta S_{m}$$ (9)

#### 4. The uniqueness constraints

The uniqueness involves two aspects. One is that after completing a trip task, each train-set should either undertake another trip or go for maintenance. The other one is that for each trip in a given train graph, only one train-set can be allocated to complete the task.

The binary variable $x_{ij}^{C}$ is defined to describe whether the trip node *i* and trip node *j* are connected by the connection arc. The binary variable $x_{ik}^{Q}$ is defined to describe whether the trip node *i* and maintenance node *k* is connected by the maintenance arc. The binary variable $x_{ki}^{H}$ is defined to describe whether the maintenance node *k* and trip node *i* are connected by the arc after maintenance. If the two nodes are connected by arcs, the value of the binary variable is 1, otherwise 0, as shown in Eqs (10)–(12).

$$x_{ij}^{C}=\{\begin{array}{ll} 1 & trip\;node\;i\;and\;trip\;node\;j\;is\;connected\;\\ & by\;a\;connection\;arc\\ 0 & otherwise \end{array}$$ (10)

$$x_{ik}^{Q}=\{\begin{array}{ll} 1 & trip\;node\;i\;and\;ma\text{intenance }node\;k\;is\;connected\\ & by\;a\;ma\text{int}enance\;arc\\ 0 & otherwise \end{array}$$ (11)

$$x_{ki}^{H}=\{\begin{array}{ll} 1 & ma\text{intenance }node\;k\;and\;trip\;node\;i\;is\;connected\\ & by\;a\;arc\;after\;ma\text{int}enance\\ 0 & otherwise \end{array}$$ (12)

Eq (13) restrains when a train-set completing Trip *i*, it should either undertake Trip *j* or go for Maintenance *k*. Eq (14) restrains that Trip *j* should be undertaken by a train-set either just completing Trip *i* or Maintenance *k*.

$$\sum_{j=1}^{a}x_{ij}^{C}+\sum_{k=1}^{b}x_{ik}^{Q}=1\;i\neq j\;\&\;k\neq i$$ (13)

$$\sum_{i=0}^{a}x_{ij}^{C}+\sum_{k=0}^{b}x_{kj}^{H}=1\;i\neq j\;\&\;k\neq j$$ (14)

### Optimization goal

Railway passenger transport corporations aim to realize profit maximization on the premise of ensuring passengers’ life and property security. Therefore, the cost is a key factor influencing the operation managers to make decisions. Due to the high acquisition cost and maintenance cost of train-sets, it is an effective measure to improve the utilization efficiency of train-sets. Thus, to complete the same number of trip tasks in a given train graph, less train-sets are needed. In a train-set utilization plan, the required amount of train-sets is equal to the value that 1440 minutes, i.e., a cycle, divides the sum of the total connecting time and trips’ running time. The running time of all trips is a fixed value given in the train graph, so the required amount of train-sets is only determined by the total connecting time. Therefore, the optimization goal of train-set utilization plan is to minimize the total connecting time, which includes the following three parts: the connecting times of all connection arcs ($\sum_{i,j\in [1,\;a],\;i\neq j}\omega_{ij}^{C}x_{ij}^{C}$), all maintenance arcs ($\sum_{i\in [1,\;a],\;k\in [1,\;b],\;i\neq k}\omega_{ik}^{Q}x_{ik}^{Q}$), and all arcs after maintenance ($\sum_{i\in [1,\;a],\;k\in [1,\;b],\;i\neq k}\omega_{ki}^{H}x_{ki}^{H}$), as shown in Eq (15).

$$\text{min}\left(\sum_{i,j\in [1,\text{ a}],\;i\neq j}\omega_{ij}^{C}x_{ij}^{C}+\sum_{i\in [1,\text{ a}],\;k\in [1,\;b],\;i\neq k}\omega_{ik}^{Q}x_{ik}^{Q}+\sum_{i\in [1,\text{ a}],\;k\in [1,\;b],\;i\neq k}\omega_{ki}^{H}x_{ki}^{H}\right)$$ (15)

## Model solution: A two-stage approach

**Fig 10:**
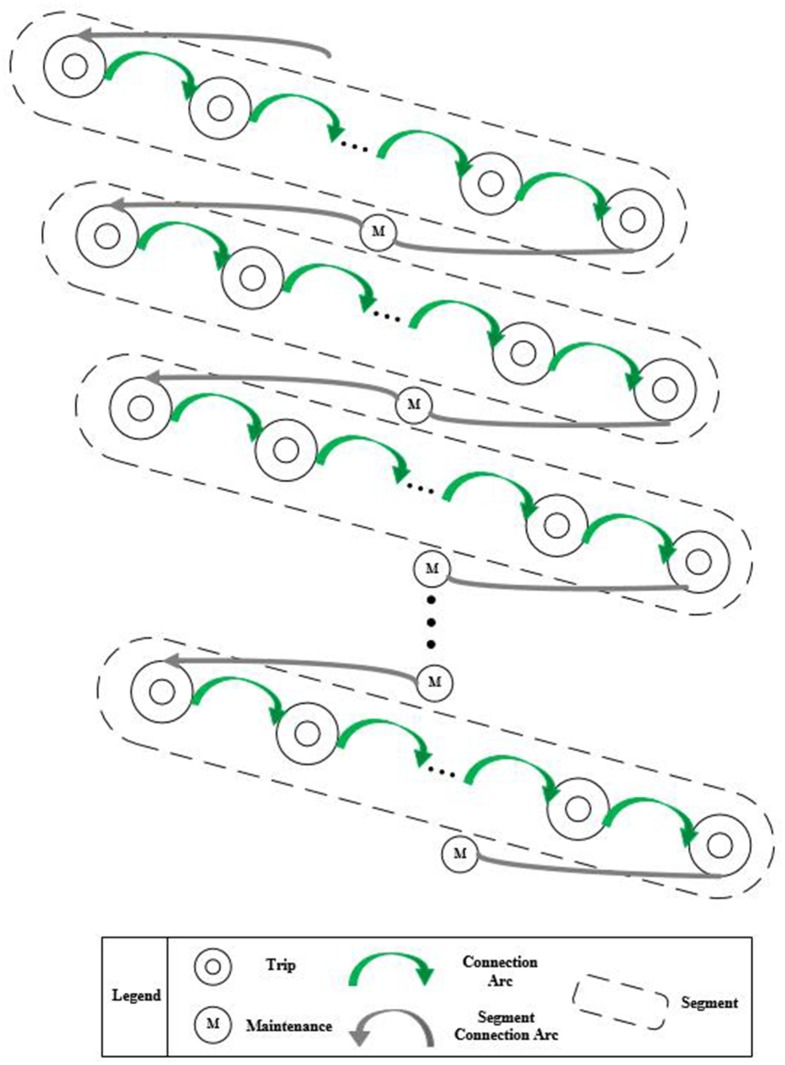
Basic process of the two-stage approach.

The train-set utilization planning model aims to identify whether arcs should be connected between trips and maintenances. Among the three kinds of arcs, the connection arcs between two trips is relatively easy to obtain due to the static characteristics that all trips are given in the train graph. Inversely, when and where the maintenances should be carried out are indeterminate according to the accumulated operation time and mileages. Therefore, the difficulty in solving the train-set utilization planning problem lies in the solving process of maintenance arcs and arcs after maintenance. To solve the above difficulty, a two stage approach is proposed. As shown in Fig 10, in the first stage, all the trips in the given train graph are allocated into a set of segments and no arcs are connected between trip nodes and maintenance nodes. In the second stage, the maintenance arcs and arcs behind maintenance will be connected between different segments. By designing a solving algorithm in each stage, the optimal and unique solution of the train-set utilization planning model can be obtained, and so the compilation of train-set utilization plan can be realized in a computer-generated system.

### The first stage: Segment generation

With the introduction of the segment concept, a directed graph $\hat{G}(\hat{V},\hat{E})$ should be defined to represent the train-set utilization network. The node set $\hat{V}$ is a union of ordered subsets ${\hat{V}}_{SE}$ and ${\hat{V}}_{M}$, which represent segments and maintenances, respectively. Each node $i\in {\hat{V}}_{SE}$ is defined as a tuple ($p_{i},f_{i}^{s},f_{i}^{t},l_{i}^{s},l_{i}^{t},time_{i},dis\text{tan}ce_{i}$), where *p*_*i*_ indicates the number of trips in the Segment *i*, $f_{i}^{s}$ and $f_{i}^{t}$ indicate the departure station and departure time of the first trip respectively, $l_{i}^{s}$ and $l_{i}^{t}$ indicate the arrival station and arrival time of the last trip respectively, *time*_*i*_ and *dis*tan*ce*_*i*_ indicate the running time and distance of all the trips respectively.

The first stage aims to obtain the connection relationship between trip nodes and prepare for the next stage simultaneously. Therefore, when allocating trips into segments, the following requirements should be met.

1. For each segment, to ensure no maintenance arcs and arcs after maintenance are included, the running time and distance of all trips should be within the maintenance standards.
2. For each segment, to ensure two adjacent trips can be connected arbitrarily, the departure station of the latter trip should be the same with the arrival station of the former trip. Moreover, the dwell time between the two trips should satisfy the requirement of operation time.

Let *ST* = {1, 2⋯, *m*} represent the set of stations, where *m* is the total number of stations in the train graph. To obtain the segment sets more efficiently, all trips are classified into a group of new trip sets based on the trips’ departure station. Let *DE* = {*DE*_1_, *DE*_2_, ⋯ *DE*_*i*_ ⋯, *DE*_*m*_} represent the above new trip sets. Particularly, all the trips in set *DE*_*i*_ ∈ *DE* departure from station *i*, where 1 ≤ *i* ≤ *m*.

Based on the above analyses, the segment generation algorithm is designed as follows.

**Algorithm 1: Segment Generation**

**while** *DE* ⊄ *ϕ*

 **do** generate segment $\hat{v}$, initialize *time* = 0, *distance* = 0;

  **for** *i* = 1 to 0

   **if** *DE*_*i*_ ⊄ *ϕ*

    add the first trip *t*_0_ in the set *DE*_*i*_ into the segment $\hat{v}$;

    *distance* = *distance* +*d*_0_, *time = time +* ($t_{0}^{a}$ − $t_{0}^{d}$);

    remove *t*_0_ from *DE*_*i*_;**break;**

   **end**

  **end**

 **while** *distance* < *S*_*m*_ + Δ*S*_*m*_ and *time* < *S*_*t*_ + Δ*S*_*t*_

  **do** find *DE*_*j*_ according to the arrival station $s_{0}^{a}$ of trip *t*_0_ in set *DE*_*i*_

   **if** *DE*_*j*_ ⊄ *ϕ*

    sort the set *DE*_*j*_ in ascending order based on the time interval between the arrival

    time of *t*_0_ in set *DE*_*i*_ and the departure time of trips in the set *DE*_*j*_

     **for** *k* = 0 to *q*_*j*_ (*q*_*j*_ denotes the number of trips in the set *ST*_*j*_)

      **if** $t_{k}^{d}-t_{0}^{a}>T_{o}$,

       *distance* = *distance* + *d*_*k*_, *time = time +* ($t_{k}^{a}$ − $t_{k}^{d}$)

       **if** *S*_*m*_ − Δ*S*_*m*_ ≤ *distance ≤ S*_*m*_ + Δ*S*_*m*_ & *S*_*t*_ − Δ*S*_*t*_ ≤ *time* ≤ *S*_*t*_ + Δ*S*_*t*_

        add *t*_*k*_ into $\hat{v}$, *t*_0_ = *t*_*k*_, remove *t*_*k*_ from *DE*_*j*_, **break**;

       **else:** *distance* = *distance*—*d*_*k*_, *time = time—*($t_{k}^{a}$ − $t_{k}^{d}$)

      **end**

     **end**

    **else**

      **break**;

 **end**

 record $\hat{v}$;

**end**

### The second stage: Segment connection

There are three decision variables in the train-set circulation planning model, including $x_{ij}^{C}$, $x_{ik}^{Q}$ and $x_{ki}^{H}$. In the first stage, the decision variable $x_{ij}^{C}$ has been solved since the connection relationships between trip nodes are obtained. Therefore, the objective of the second stage is to calculate the decision variables $x_{ik}^{Q}$ and $x_{ki}^{H}$, i.e., the connection relationship between trips and maintenance. With the concept of segment, the arcs between segments is equivalent to the combination of the maintenance arc and arc after maintenance. Therefore, the second stage aims to identify the segment connection relationship.

When node $i\in {\hat{V}}_{SE}$ is connected to node $j\in {\hat{V}}_{SE}$, it means that the train-set should be maintained after completing the last trip in Segment *i*, and then undertake the first trip in Segment *j*. Therefore, to ensure that node $i\in {\hat{V}}_{SE}$ can be connected to node $j\in {\hat{V}}_{SE}$, the following requirements should be satisfied:

1. The arrival station of the last trip, $l_{i}^{s}$ in Segment *i* should be the same with the departure station of the first trip, $f_{i}^{s}$ in Segment *j*.
2. The time interval, $\omega_{ij}^{S}$, between the last trip in Segment *i* and the first trip in Segment *j* should be longer than the duration of a series of working procedures, which is equal to the sum of *T*_*Z*_ and *T*_*C*_. Particularly, the value of $\omega_{ij}^{S}$ can be obtained by Eq (16).

$$\omega_{ij}^{S}=\{\begin{array}{ll} f_{j}^{t}-l_{i}^{t} & f_{j}^{t}-l_{i}^{t}\geq T_{Z}+T_{C}\\ 1440+f_{j}^{t}-l_{i}^{t} & f_{j}^{t}-l_{i}^{t}<T_{Z}+T_{C} \end{array}$$ (16)

**Algorithm 2: Segment Connection**

Step 1 Initial segment node set ${\hat{V}}_{SE}=\{{\hat{v}}_{1},{\hat{v}}_{2}\cdots ,{\hat{v}}_{n}\}$, arc set *E* = *ϕ* and station set *ST* = {1, 2⋯, *m*}, let *st* = 1.
Step 2 Classify the segment set ${\hat{V}}_{SE}$ according to the last trip arrival station, $l_{i}^{s}=st$, and the first trip departure station, $f_{j}^{s}=st$; get arrival segment set ${\hat{V}}_{st}^{a}=\{{\hat{v}}_{i}|{\hat{v}}_{i}=(p_{i},f_{i}^{s},f_{i}^{t},l_{i}^{s}=st,l_{i}^{t},time_{i},dis\text{tan}ce_{i})\}$ and departure segment set ${\hat{V}}_{st}^{d}=\{{\hat{v}}_{j}|{\hat{v}}_{j}=(p_{j},f_{j}^{s}=st,f_{j}^{t},l_{j}^{s},l_{j}^{t},time_{j},dis\text{tan}ce_{j})\}$, sort the two sets by time ascending.
Step 3 Calculate $\omega_{ij}^{S}$ according to the above rule, get the matrix {$\omega_{ij}^{S}$}.
Step 4 Gain a set of optimal solutions by the Hungarian Algorithm, get the matrix {$\omega^{\prime }{}_{ij}^{S}$}, add the zero corresponding arc into set *E*.
Step 5 If *st* = *m*, end the algorithm; else let *st* = *st* + 1 and go to step 1.

**Fig 11:**
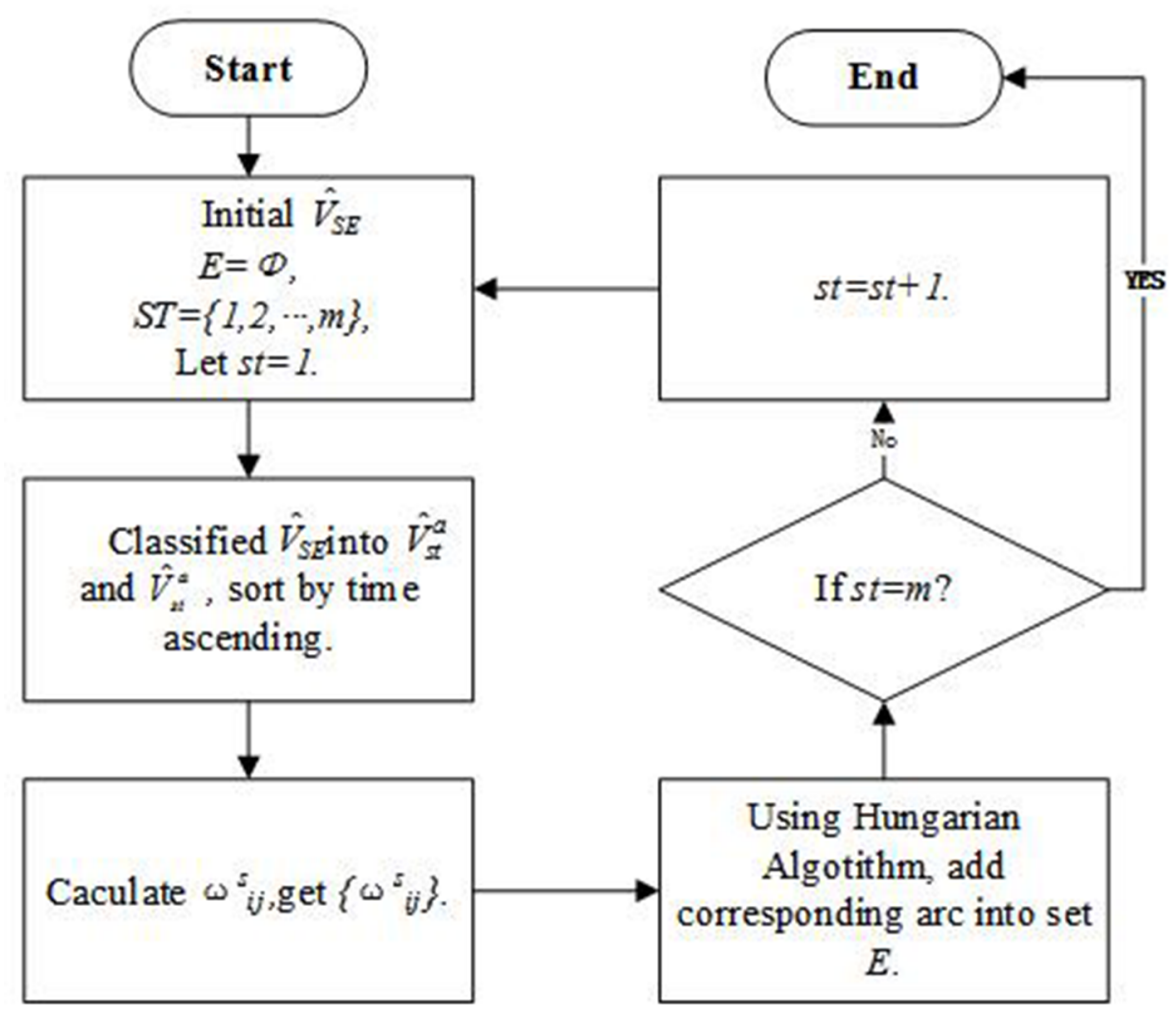
Flow chart of the segment connection.

The segment connection algorithm is designed based on the Hungarian algorithm. The flow chart is shown as Fig 11.

## Case studies

In this section, the case of the train graph of Beijing-Tianjin passenger dedicated line was adopted to test the performance of the proposed train-set utilization model and solution approach. Several evaluation indexes were applied to evaluate the quality of the computation results. Furthermore, the comparison between the ant colony algorithm and the two-stage approach was conducted to evaluate the advancements. The proposed approach is coded in C# and solved by Microsoft Visual Studio, and the computational experiments were performed on a 3.4 GHz Core i7 PC with 16 GB of RAM. The proposed model can be solved within a few seconds.

### Main features of the case study

**Fig 12:**
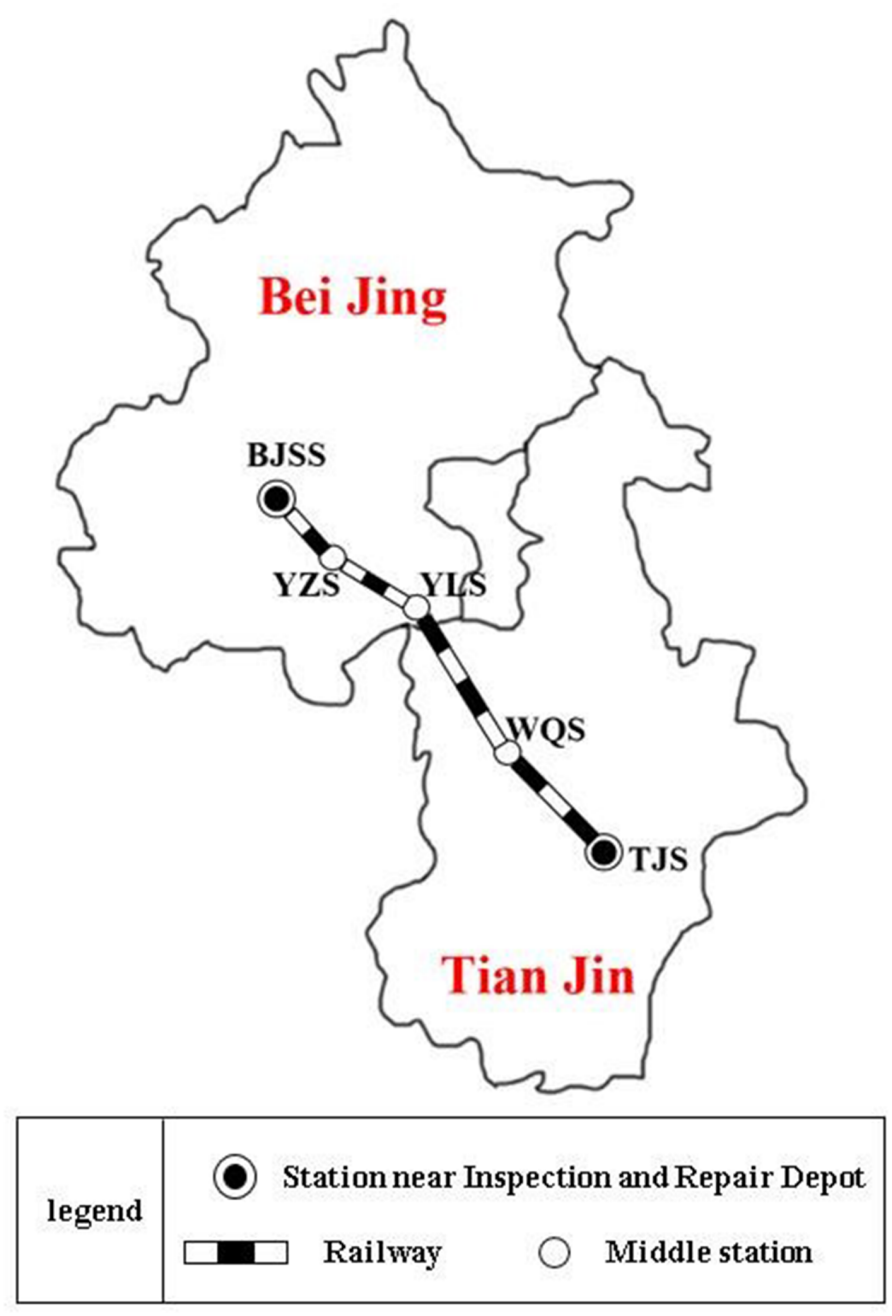
The topological structure of Beijing-Tianjin passenger dedicated line.

Fig 12 illustrates the topological structure of the Beijing-Tianjin passenger dedicated line, the length of which is nearly 120 kilometers. There are five stations along the line, including two endpoint stations (Beijing South Station (BJSS) and Tianjin Station (TJS)), and three middle stations (Yizhuang Station (YZS), Yongle Station (YLS), and Wuqing Station (WQS)). Moreover, only the two endpoint stations BSS and TJS are located near the repair and inspection depot. Therefore, the maintenance of train-sets can be carried out only after arriving at BJSS and TJS.

**Fig 13:**
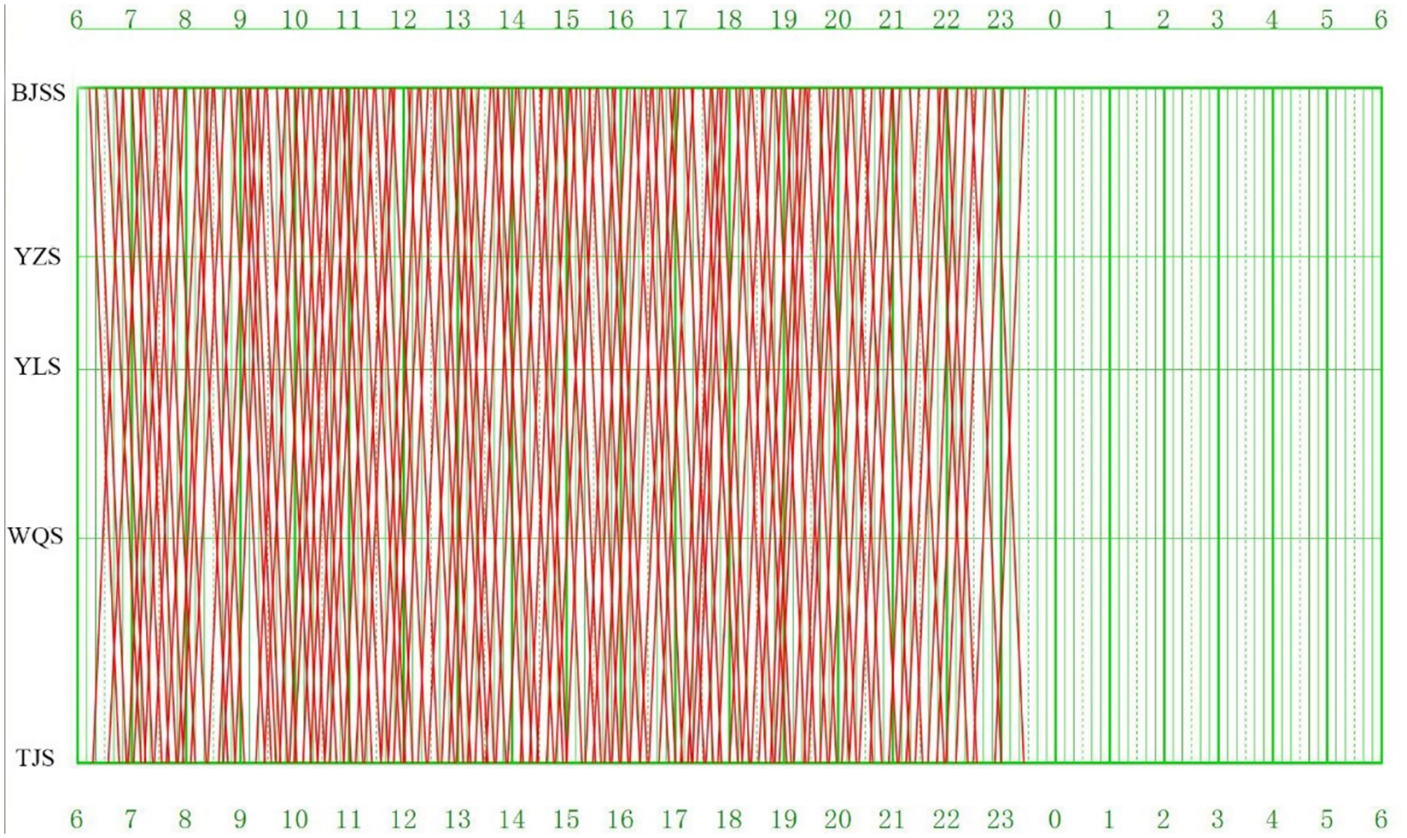
Train graph of Beijing-Tianjin passenger dedicated line.

Actually, the train graph used in this study was carried out in December, 2014. As shown in Fig 13, there are 174 trips in total in the train graph, among which 86 trips are from BJSS to TJS and 88 trips are from TJS to BJSS. The total running distances of all the trips, *D*_*all*_, is equal to 20,880 kilometers, and the total running time, *T*_*all*_, is equal to 6,090 minutes.

The parameters related to the model and approach are listed as follows:

- Basic operation time *T*_*o*_: $T_{o}^{BJSS}=14$ minutes; $T_{o}^{TJS}=14$ minutes.
- Minimal duration of the four essential procedures if train-sets needs to be maintained *T*_*Z*_: $T_{Z}^{BJSS}=261$ minutes; $T_{Z}^{TJS}=257$ minutes.
- Duration of preparation work before train-set departing in station *T*_*C*_: $T_{C}^{BJSS}=T_{C}^{TJS}=28$ minutes.
- Distance range of maintenance standard *S*_*one*_: 3, 600 *kilometers* ≤ *S*_*one*_ ≤ 4, 400 *kilometers*.
- Time range of maintenance standard *T*_*one*_: 1.8 *day* ≤ *T*_*one*_ ≤ 2.2 *day*.

### Computational results of the train-set utilization plan

**Fig 14:**
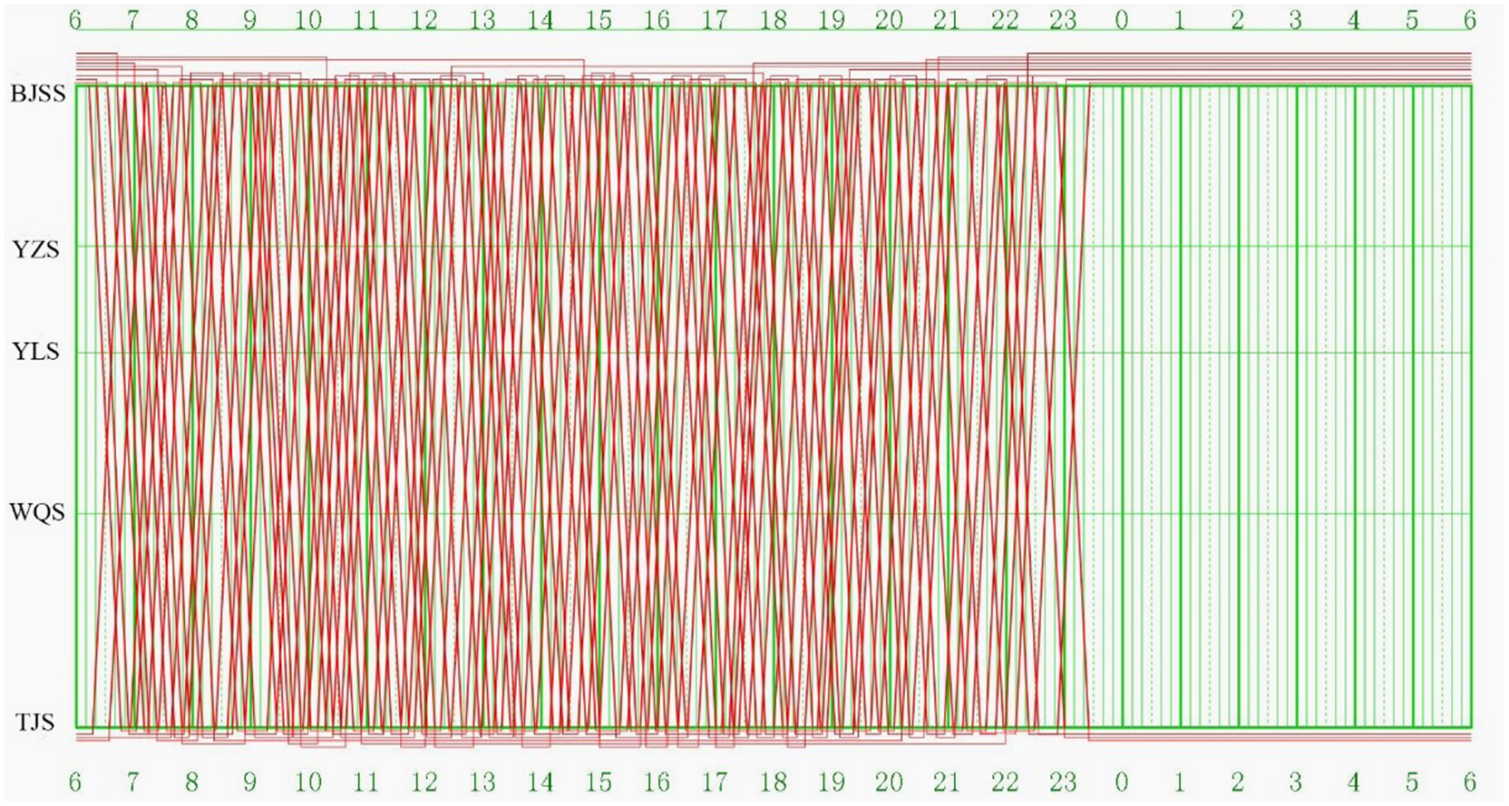
Train-set utilization plan in Beijing-Tianjin passenger dedicated line.

Fig 14 illustrates the computational results of the model, i.e., the train-set utilization plan of the train graph of Beijing-Tianjin passenger dedicated line carried out in December, 2014. Table 3 lists the results of some important evaluation indexes.

**Table 3:** Computational results.

Train-sets Amount	Maintenance Times	Utilization Rate	Connecting Time
12	6	46.9%	31,055 minutes

The results show that to complete the above 174 trip tasks, the minimal amount of needed train-set *N* is 12. The total maintenance times *M* of all the 12 train-sets is 6 each day. The average running distances of each train-set per day *D*_*average*_ = *D*_*all*_ / *N* is 1740 kilometers. Each day, the rail line overhaul is carried out during the period 0:00–6:00. Therefore, the train-sets cannot work for six hours per day, and the maximal running time of each train-set can be calculated as follows: 1440–6 * 60 = 1080 minutes. The average utilization efficiency of each train-set *U* = *T*_*all*_ / (1080 * *N*) reaches 46.9%.

Theoretically, the minimum connection time of this case is obtained by applying Hungarian algorithm, which is equal to 25,542 minutes when maintenance constraints are not considered. Moreover, the total maintenance times of all train-sets is 4.7 each day, which can be calculated as *D*_*all*_ / (1 + 10%)*S*_*one*_. However, the theoretical results cannot be reached in practical production, because train-sets must be maintained according to the rules and regulations. Also, not all train-sets need to be maintained until their running distances reaching the upper limit of the standard. Therefore, the connection time 31,055 minutes and maintenance times 6 obtained by the proposed model and approach are very close to the theoretical values. These results prove that the proposed model and approach are feasible and the train-sets are utilized with high efficiency.

### Comparison with the ant colony algorithm

Many researchers have applied the ant colony algorithm (ACA) to solve train-set utilization problem. In order to highlight the performance of this study in improving the train-set utilization efficiency, the ant colony algorithm is applied to carry out the same experiments with the same computer, and the detailed comparison results are as listed in Table 4. The results show that obviously the two-stage approach has the advantages over the traditional ACA in improving the solution quality and shortening the computation time.

**Table 4:** Results of the comparison between the ant colony algorithm and the two-stage approach.

	Two-stage approach	Traditional ACA	Difference
*N*	12	13	1
*M*	6	8	2
*D*_*average*_ (kilometers/day)	1740	1606	134
*U*	46.9%	43.4%	3.5%
Computational time (millisecond)	2302	8032	5730

On one hand, the utilization efficiency of train-sets is much higher by applying the two-stage approach. First, the amount of train-sets in use is reduced, which will save the acquisition cost significantly. Second, the total maintenance times is decreased from eight to six, saving much invalid running time from the railway stations to the inspection and repair depot. Third, the average running distances of each train-set per day is 134 kilometers longer than that of the traditional ACA.

On the other hand, the computation time of the two-stage approach is 2302 milliseconds, which is almost one-third of the traditional ACA. What should be noted is that, as the convergence speed of the traditional ACA is indeterminate, an average of 20 computation times is used. Furthermore, the computation of the two-stage approach is much more stable. Only one optimal train-set utilization plan can be obtained with two-stage approach while different results are achieved by using the traditional ACA.

There are certain scope and limitations in the study. Firstly, the proposed approach is applicable for a short-term train-set circulation plan formulation. In China, there are five levels of maintenances in total, and the maintenance of level one is considered only in the study. As the maintenance of Level 2 to Level 5 take more than one month usually, it is beyond the scope of consideration in the study. Secondly, only one type of train-sets is studied by using the proposed approach, so the approach is appropriate for the new-built HSR with single type train-set, instead of the multi-type train-sets situation. As China has developed standard train-sets and tried to use the standard train-set as the main carrier in the future, the present approach will show important referential meaning in the utilization of standard train-set in the future.

## Conclusion

The train-set utilization plan is drawn up to identify the work arrangements of train-sets, which is profoundly affected by the given train graph as well as the rules and regulations of maintenance. To optimize the train-sets’ utilization efficiency, in this study, an integer programming model is proposed considering the spatial constraints, the time constraints, the maintenance constraints, and the unicity constraints. In the process of model solution, a two-stage approach is designed and an optimal train-set utilization plan is obtained as the output. Using the model, the case of the real train graph of Beijing-Tianjin passenger dedicated line was carried out. The results show that the model and approach proposed in this study is practical and reasonable. By comparing the results obtained by the two-stage approach and the ant colony algorithm, it could be found that to complete the same trip tasks, the former one needs less train-sets, less maintenance times, and longer average running distances for each train-set per day. In short, the utilization efficiency of train-sets is much higher by applying the two-stage approach. Moreover, the fast computation speed and good stability can contribute to the realization of a train-set utilization plan computer-generated system.
